# Intrathecal administration of mesenchymal stem cells in patients with adrenomyeloneuropathy

**DOI:** 10.3389/fneur.2024.1345503

**Published:** 2024-02-02

**Authors:** Tomasz Siwek, Beata Zwiernik, Katarzyna Jezierska-Woźniak, Kamila Jezierska, Marcin P. Mycko, Krzysztof W. Selmaj

**Affiliations:** ^1^Department of Neurology, University of Warmia and Mazury in Olsztyn, Olsztyn, Poland; ^2^University Hospital, University of Warmia and Mazury in Olsztyn, Olsztyn, Poland; ^3^Laboratory for Regenerative Medicine, Department of Neurosurgery, University of Warmia and Mazury in Olsztyn, Olsztyn, Poland; ^4^Center of Neurology, Lodz, Poland

**Keywords:** adrenomyeloneuropathy, mesenchymal stem cells, motor function, WJ-MSC, AMN

## Abstract

**Background and objectives:**

X-linked adrenomyeloneuropathy (AMN) is an inherited neurodegenerative disorder associated with mutations in the ABCD1 gene and the accumulation of very long-chain fatty acids (VLFCAs) in plasma and tissues. Currently, there is no effective treatment for AMN. We have aimed to evaluate the therapeutic effects of mesenchymal stem cell (MSC) transplantation in patients with AMN.

**Methods:**

This is a small cohort open-label study with patients with AMN diagnosed and treated at the University Hospital in Olsztyn, Poland. All patients met clinical, biochemical, MRI, and neuropsychological criteria for AMN. MSCs derived from Wharton jelly, 20 × 10^6^ cells, were administered intrathecally three times every 2 months, and patients were followed up for an additional 3 months. The primary outcome measures included a blinded assessment of lower limb muscle strength with the Medical Research Council Manual Muscle Testing scale at baseline and on every month visits until the end of the study. Additional outcomes included measurements of the timed 25-feet walk (T25FW) and VLFCA serum ratio.

**Results:**

Three male patients with AMN with an age range of 26–37 years participated in this study. All patients experienced increased muscle strength in the lower limbs at the end of the study versus baseline. The power grade increased by 25–43% at the baseline. In addition, all patients showed an improvement trend in walking speed measured with the T25FW test. Treatment with MSCs in patients with AMN appeared to be safe and well tolerated.

**Discussion:**

The results of this study demonstrated that intrathecal administration of WJ-MSC improves motor symptoms in patients with AMN. The current findings lend support to the safety and feasibility of MSC therapy as a potentially viable treatment option for patients with AMN.

## Introduction

Adrenomyeloneuropathy (AMN) and cerebral adrenoleukodystrophy (CALD) are the clinical forms of X-linked adrenoleukodystrophy (X-ALD), an inherited progressive neurometabolic disease caused by mutations in the ABCD1 gene and the accumulation of very long-chain fatty acids in cells and tissues (1). AMN symptoms include spastic paraparesis, sensory ataxia with impaired vibration sense, and signs of Addison disease in 50–70% of patients (2, 3). Patients with AMN, contrary to cerebral ALD (CALD), have no MRI brain lesions, unremarkable spinal cord imaging, and intact cognitive functions (4–6). Peripheral neuropathy may also be present; however, it is usually masked by the dominant signs of myelopathy (7). The disease is non-invertible progressive and significantly reduces life expectancy. There is no known cure for AMN. Several therapies have been tried, including Lorenzo Oil (8), bone marrow transplantation (9), hematopoietic stem cell transplantation (10–12), antioxidants (13), and statins (14), with very limited efficacy. The most recent study with leriglitazone, a novel selective peroxisome proliferator-activated receptor gamma agonist, also did not meet the primary endpoint (15).

Mesenchymal stem cells (MSCs) represent one of the major categories of human stem cells with moderate differentiation capabilities but the ability to repair damaged tissues and organs (16). The clear advantage of MSCs over other stem cells, induced pluripotent stem cells, and embryonic stem cells is their lack of ethical issues and lack of risk for cancer. MSCs can be derived from various human tissues, such as bone marrow, adipose tissue, umbilical cord, skin, and muscles (17). They can differentiate into mesoderm-derived tissues such as bone, cartilage, blood vessels, and cardiomyocytes, as well as ectoderm-derived neurons and glial cells (18). The primary role of MSCs during adult life is to repair and replace damaged tissue. MSCs can be administered in an autologous setting with no risk of immunological rejection. Thus, MSCs represent a very promising method of treatment in regenerative medicine. Inspired by reports on encouraging results of MSC therapy in demyelinating and neurodegenerative conditions (15, 19, 20) including our own experience with ALS (21) and experimental models of MS^22^, we have aimed to evaluate the efficacy of intrathecal administration of MSCs derived from Wharton jelly (WJ) in three patients with AMN.

## Methods

### Patients, inclusion, and exclusion criteria

The study was performed at University Hospital in Olsztyn, Poland. The inclusion and exclusion criteria for the study were applied. Inclusion criteria involved adult age, diagnosis of AMN confirmed with the results of an increased VLCFA serum ratio (Table 1), and lower limb spastic paraparesis. Exclusion criteria involved other causes of spastic paraparesis, assessed clinically and by spinal MRI, and the presence of CALD in the form of MRI brain lesions or cognitive dysfunctions in psychological tests. Three male patients, aged 33, 37, and 26 years, met the criteria and were enrolled in the study.

**Table 1:** Serum VLCFA 24:22 and VLCA 26:22 ratio at baseline.

	Patient 1	Patient 2	Patient 3	Laboratory standards for ALD individuals
VLCFA 24:22 ratio	1.552	1.823	1.75	>1.0
VLCFA 26:22 ratio	0.04	0.025	0.049	>0.02

### Standard protocol approvals, registrations, and patient consents

All patients have signed the informed consent form, and all procedures of this study were approved by the Bioethical Committee of the University of Warmia and Mazury in Olsztyn, Poland. Number of approval: 10/2017.

### MSC preparation

The human umbilical cord was obtained aseptically from a full-term uncomplicated pregnancy after the completion of a planned cesarean section. Wharton jelly MSCs (WJ-MSCs) were isolated using the explant isolation method, as described previously with minor modifications (22). After immersion in a sterile vessel containing 0.01 M phosphate-buffered saline (PBS, pH 7.2) supplemented with 1% penicillin–streptomycin (10,000:10,000; Sigma-Aldrich, St. Louis, MO, United States), the cords were cut into small pieces (1–2 cm in length) and transferred to 60 × 15 mm Petri dishes, containing DMEM/F-12, GlutaMAX supplemented with 1% P/S, and 10% fetal bovine serum (FBS; Sigma-Aldrich), and incubated at 37°C in a humidified atmosphere containing 5% CO_2_ for future culture. Before intrathecal administration, the cells were maintained in DMEM/F-12 medium without serum, using only a single passage. Subsequently, cells were detached and washed three times with PBS 1× and once with autologous cerebrospinal fluid. All these procedures were performed according to the GMP grade, including sterility, microbiology, endotoxin testing, and karyotype stability.

### MSC characterization

The isolated MSCs were characterized by phenotyping with MSC-specific cell surface markers using the BD Stemflow-Human MSC Analysis Kit (BD Biosciences, Franklin Lakes, NJ, United States), according to the International Society for Cellular Therapy guidelines (23). Flow cytometry was performed using a fluorescence-activated cell sorter (BD FACS Aria II, BD Biosciences, Franklin Lakes, NJ, United States), and the results were analyzed with DIVA software. The obtained MSCs fulfilled the International Society for Cellular Therapy criteria (23), including a phenotypic characterization: CD105/CD73/CD90/CD44 positive and CD45/CD34/CD11b/CD19/HLA-DR PE negative.

### MSC administration

Cells were injected intrathecally by standard lumbar puncture procedure with an amount of 20 × 10^6^ cells in a volume of 2 mL. MSCs were suspended in autologous cerebrospinal fluid. Two patients received three injections every 2 months, while the third patient received two injections. Patients were followed up for 7 months (one patient up to 4 months).

### Neurological evaluation

Patients were neurologically examined prior to MSC administration, and each subsequent visit was 1 month apart until visit 7, with one patient examined until visit 4. The muscle strength of the lower limbs was assessed with the Medical Research Council Manual Muscle Testing scale (24). We assessed the strength of seven muscle groups of the lower limbs: hip flexors, hip extensors, hip adductors, knee flexors, knee extensors, foot flexors, and foot extensors in both legs. The grade (0–5) was assessed for each muscle group, and the mean for both legs was used as a final score. A timed 25-feet walk (T25FW) was performed twice at each visit, and the mean results were used for further analysis.

### Laboratory tests

The serum ratios of VLCFA 24:22 and VLCFA 26:22 were assessed by gas chromatography (GC) technique as described before (25) at every other month.

### MRI

All patients had MRI examinations of the brain, cervical, and thoracic spines. MRI data were acquired on a 3.0 T scanner (Siemens, Erlangen, Germany). MRI images used for this study were obtained ±2 weeks from MSCs first administration. To detect focal white matter lesions, MRI used dual-echo (repetition time [TR] = 4,500 ms, echo time [TE] = 22 and 90 ms, 25 slices, slice thickness = 3 mm, 512 × 512 × 44 matrix, field of view [FOV] = 250 mm), and T1-weighted (TR = 750 ms, TE = 17 ms, 25 slices, slice thickness = 3 mm, 512 × 512 × 44 matrix, FOV = 250 mm). Gadolinium (Gd)-enhancing T1-weighted lesions were identified from post-contrast T1-weighted spin echo images (TR = 467 ms, TE = 8 ms, 240 × 240 × 132 mm) FOV (number of excitations = 1), acquired 5 min after administration of a dose of contrast (0.1 mM/kg). All MRI images were obtained at baseline and at the end of the study.

### Neuropsychological tests

The neuropsychological evaluation included a battery of cognitive function tests. All patients were assessed for visual–spatial functions, attention and visual inspection, mental work pace, executive functions, planning, and abstract thinking derived from visual–spatial stimuli. Mini-Mental State Examination (MMSE), the Combination Test of Points A and B, the Clock Drawing Test, and Benton’s Visual Remembrance Test (BVRT) were applied to all patients before MSC administration and at the end of the observation period.

### Statistical analysis

Statistical analysis for muscle strength parameters, T25FW, and VLCFA ratios was performed with non-parametric Mann–Whitney tests. *p* < 0.05 were considered statistically significant.

## Results

### Safety of MSC intrathecal administration

After intrathecal MSC administration, all patients experienced modest transient side effects typical of post-lumbar puncture syndrome, such as headache and mild nausea, for 1–4 days. All these symptoms resolved spontaneously or required treatment with paracetamol. None of the patients have reported any serious side effects through the course of follow-up observation.

### MSC administration effect on muscle strength

**Figure 1:**
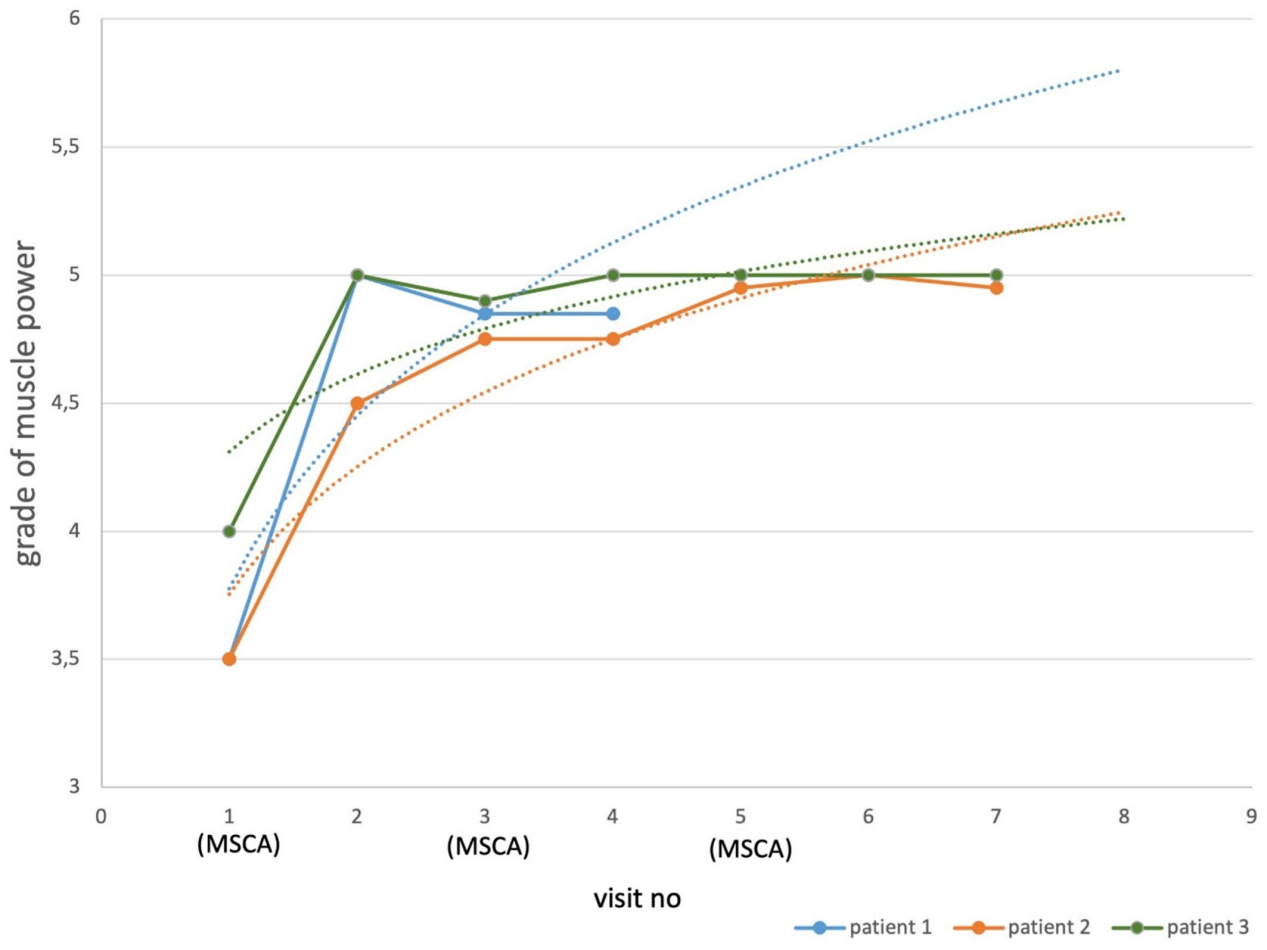
Muscle power in AMN patients treated with MSCs. Each point represents a mean grade of muscle strength of lower limbs for individual visit. Dotted lines, made by logarithmic functions, demonstrate an improvement trend for each patient. The difference in muscle strength grades between baseline and the last visit was significant (*p* = 0.04) for all three patients.

The mean grade of muscle strength of seven groups of muscles in both lower limbs showed a marked increase after the first MSC administration in all three patients (Figure 1). An improvement of the mean grade of muscle strength after the first MSC administration versus baseline for patient no. 1 was 1.5/3.5 points (43%), for patient no. 2, 1/3.5 points (29%), and for patient no. 3 1/4 points (25%) The improvement in muscle strength has been maintained in all three patients until the last follow-up visit (Figure 1). The improvement of muscle strength for all three patients on the last visit versus baseline was statistically significant (*p* = 0.04; Figure 1).

### MSC administration effect on T25FW

**Figure 2:**
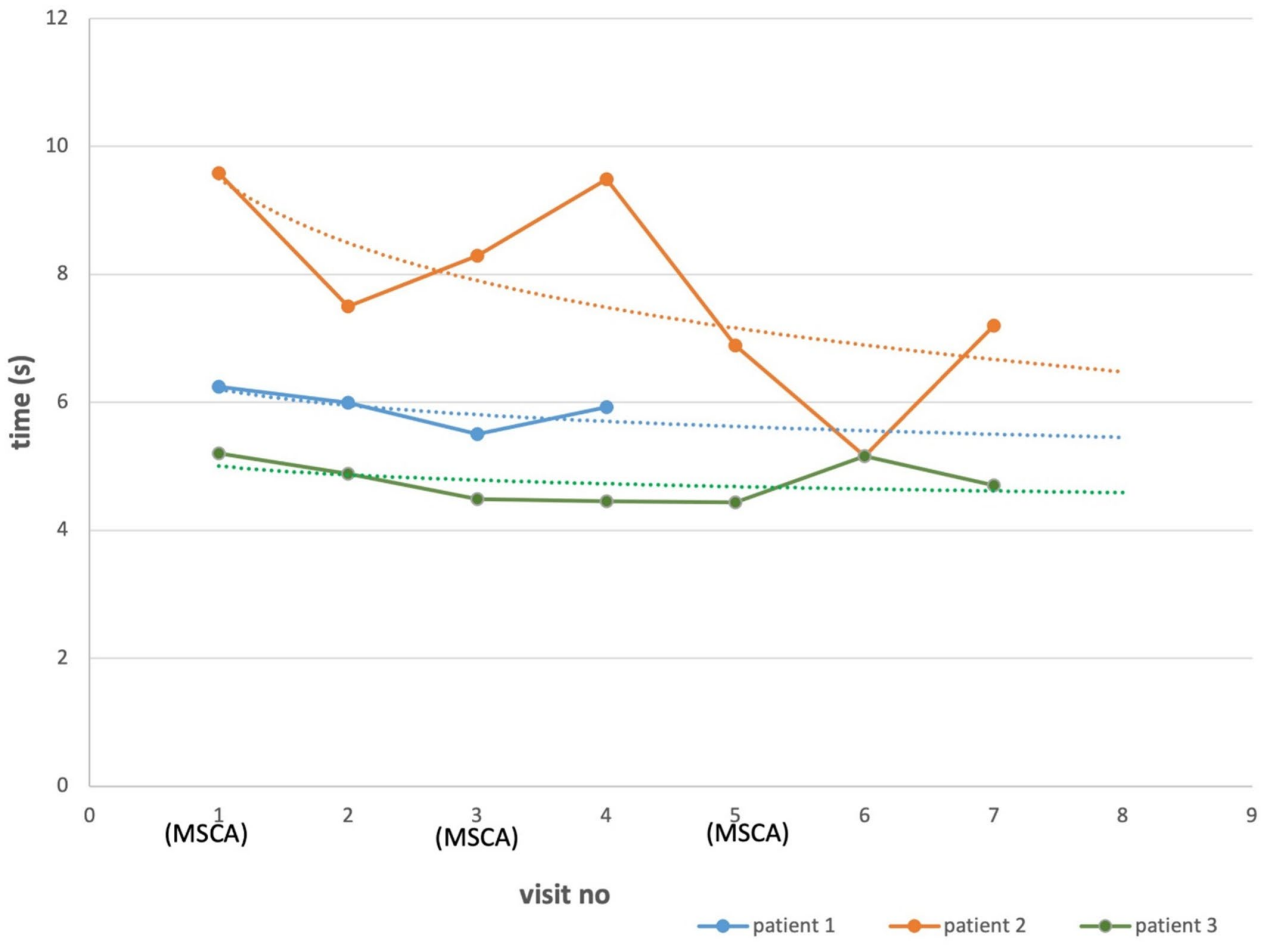
T25FW in AMN patients treated with MSCs. Each point represents a mean of two recordings of the same patient. Dotted lines, made by logarithmic functions, demonstrate an improvement trend for each patient. The difference between baseline and the last visit measurements were not statistically significant (*p* = 0.51).

All three patients showed decreased T25FW after the first MSC administration versus baseline, indicating improvement in walking speed (Figure 2). The T25FW on the last follow-up visits was shorter than the baseline for all three patients. However, the difference between baseline values and each of the subsequent measurements was not statistically significant (*p* = 0.51).

### MSC administration effect on VLCFA ratio in serum

**Figure 3:**
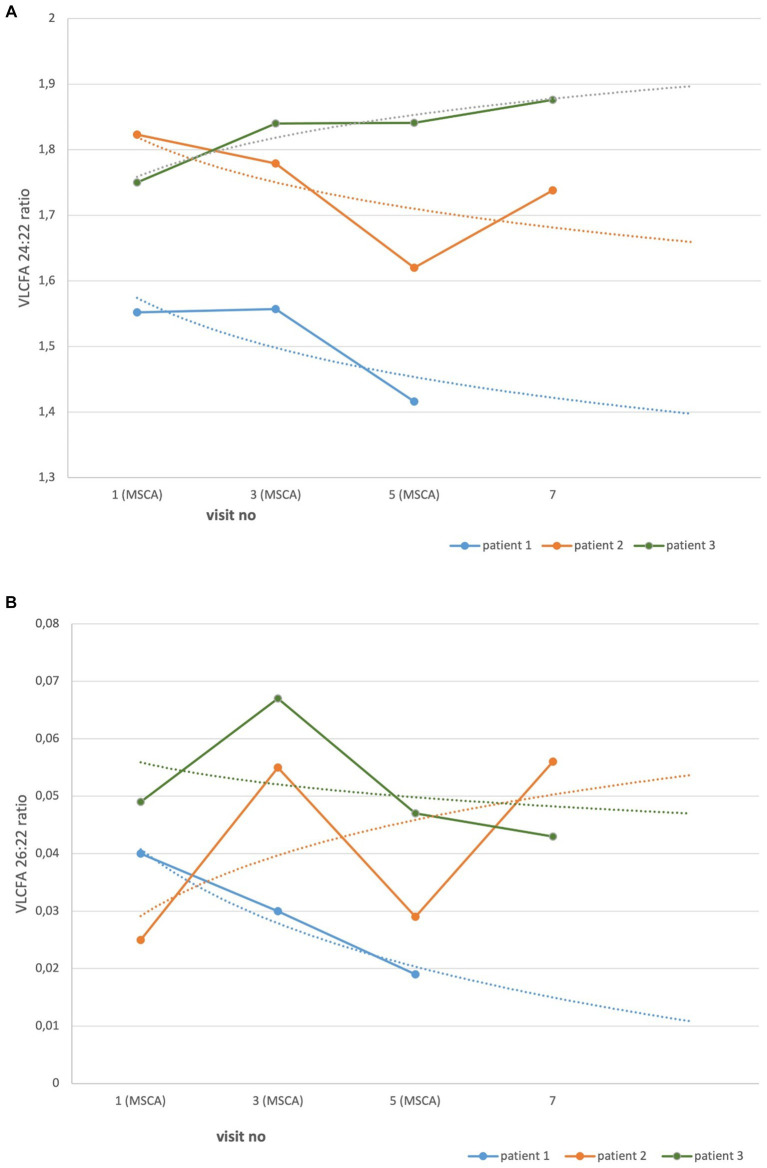
VLCFA serum ratio in AMN patients treated with MSCs. Each point represents the serum ratio of VLCFA. Dotted lines, made by logarithmic functions, demonstrate a trend in all patients. The difference between baseline and the last follow-up visit measurements were not statistically significant. **(A)** VLCF 24:22, *p* = 0.81. **(B)** VLCF 26:22, *p* = 0.82.

At baseline visit, VLCFA showed abnormalities pathognomonic for ALD (Table 1). The serum ratio of the VLCFA 24:22 measured at follow-up visits versus baseline decreased in two patients and increased in one. The difference between the ratio of baseline and the last follow-up visit was not significant (*p* = 0.81). Similarly, the VLCFA 26:22 ratio decreased in two patients and increased in one. However, the difference between the baseline and the last follow-up visit was not significant (*p* = 0.82; Figures 3A,B).

### MRI

Patients did not have demyelinating brain and spinal cord lesions at baseline. At the last follow-up visit, there were no changes in brain and spinal cord MRI images.

### Cognitive functions

Neuropsychological parameters did not reveal any deficiency in the scope of cognitive skills at baseline and subsequent follow-up visits, as expected in AMN (Table 2).

**Table 2:** Neuropsychological test results.

		Visit 1 (MSCA)	Visit 3 (MSCA)	Visit 5 (MSCA)
Patient 1	BVRT	10	10	
	MMSE	29	29	
	Combination test of Points A and B	26 s/52 s	18 s/54 s	
	The Clock Drawing Test	10	10	
Patient 2	BVRT	8	9	9
	MMSE	30	30	30
	Combination test of Points A and B	23 s/50 s	20 s/50 s	23 s/49 s
	The Clock Drawing Test	10	10	10
Patient 3	BVRT	9	10	10
	MMSE	29	30	30
	Combination test of Points A and B	25 s/55 s	24 s/57 s	21 s57 s
	The Clock Drawing Test	10	10	10

MMSE, Mini-Mental State Examination; BVRT, Benton’s Visual Remembrance Test.

### Classification of evidence

The aim of the study was to determine the effect of intrathecal MSC administration on the symptoms of patients with AMN. Three patients participated in the study and showed improvement in the strength of the muscles of the lower limbs and improvement in walking speed. Due to the small number of participants, this study should be identified as Class IV of the Classification of Evidence.

## Discussion

The results of this study showed that the intrathecal administration of allogeneic WJ-MSC in three AMN patients produced an improvement in the muscle strength of the lower limbs. However, the MSC effect on T25FW showed only a positive but not significant trend. In addition, the VLCFA serum ratio in two out of three patients showed transient, but not significant decrease after intrathecal infusions of MSCs.

There is no approved treatment for ALD. In early cases of CALD, allogenic hematopoietic stem cell transplantation (HSCT) is recommended to prevent disease progression and long-term stabilization (26, 27). Although the direct mechanism of HSCT activity is not clear, it is assumed that transplanted HSCs deliver the cellular source of the intact ABCD1 gene and corrected VLCFA ratio (12). It was shown that HSCs cross the blood–brain barrier and modify microglia in the brain (28). The clinical effects of HSCT are usually evident 6 months post-transplantation. An alternative approach is gene therapy with autologous HSCs transfected with the wild-type ABCD1 gene (29). It should be remembered, however, that allogeneic HSCT remains associated with significant morbidity and mortality risks. Thus, before applying HSCT to ALD patients, the ratio of benefit versus risk should be assessed. HSCT has not been tested systematically in AMN.

MSCs are known to express neurotrophic and neuroprotective activity and contribute to tissue repair in several conditions (30). It has been repeatedly shown that MSCs ameliorate symptoms in numerous experimental models and in patients with neurodegenerative disorders, including multiple sclerosis. MSCs transferred to mice with experimental autoimmune encephalomyelitis (EAE), a model of multiple sclerosis, enhanced recovery, prevented relapses, and promoted myelin repair (31). Several mechanisms were attributed to the beneficial effects of MSCs in demyelinating conditions, including trophic support, immunomodulation, and metabolic signaling (32). Human MSCs transplanted in EAE mice migrated to the CNS and supported amelioration of clinical symptoms (33). The effect of MSCs on human demyelinating disorders is less evident because of the lack of large control studies. Most data are coming from small trials of the 1 or 1/2 phases. Despite that, several of these studies did demonstrate the beneficial effect of MSCs in patients with multiple sclerosis (20, 34). These beneficial effects were usually seen from a short-time perspective, but in one study, long-term stabilization of the clinical course of remitting relapsing MS was observed for 4 years (35). In addition, MSCs showed an effect on disability improvement in a 6-month trial (36). Similarly, in secondary progressive MS patients, MSC treatment resulted in diminished MRI progression (37). Recently, it was shown in progressive MS patients that MSC-induced neurotrophic factor secretion improved motor activity and increased levels of neurotrophic factors, parallel to diminishing proinflammatory mediators in the CSF (38). The results of studies assessing MSC neuronal progenitors demonstrated improvement in muscle strength and adequately diminished EDSS in 70% of MS patients (39).

The rationale to apply MSCs for the treatment of ALD is supported by the observation that MSCs secrete factors that enhance axonal outgrowth and neuronal cell survival. MSCs promoted neurogenesis and axonal sprouting, contributing to cell differentiation, replacement, and integration within CNS (40). Of particular relevance might be the observation that MSCs from bone marrow express alpha-L-iduronidase, arylsulfatase-A and B, glucocerebrosidase, and adrenoleukodystrophy protein (41). However, until now, there has only been one study on the use of MSCs in ALD. The results of this study did not support beneficial effects on ongoing disease activity in two patients. These results are in contrast to our findings about increased muscle strength in lower limbs in three AMN patients treated with intrathecal WJ-MSC for 4–7 months. In the ALD study, MSCs were administered only once for one patient and three times, with short intervals 1 week apart, in the second patient. The follow-up period was limited to 1 and 2 months, and the readout involved only MRI endpoints. The dose of MSCs involved in the ALD study was also much lower than the dose used by us, 5 × 10^6^/kg versus 20 × 10^6^, respectively. When comparing the results of these two studies, the mechanistic differences between CALD and AMN resulting from an interplay between genetic and environmental factors should be considered. Despite being a monogenetic disease, mutations in the ABCD1 gene have no predictive value with respect to clinical outcomes. Accordingly, we have not seen any effect of MSCs on serum VLCFA. The lack of a simple genotype–phenotype correlation can be exemplified by the presence of several clinical types of ALD. AMN is a significantly less inflammatory condition than CALD, with no brain MRI lesions and no or minimal MRI lesions in the spinal cord. AMN is also significantly less progressive compared to CALD (42). The intrathecal administration of WJ-MSC to AMN patients was safe and well tolerated, with only minor side effects typical for post-lumbar puncture syndrome.

In summary, the data from this study demonstrated that the intrathecal administration of WJ-MSC improves motor strength in AMN. The current findings lend support to the safety and feasibility of MSC therapy as a potentially viable treatment option for patients with AMN.

## Author’s note

Due to low number of participants, this study fulfills the criteria for class IV of the Classification of Evidence.

## Data availability statement

The raw data supporting the conclusions of this article will be made available by the authors, without undue reservation.

## Ethics statement

The studies involving humans were approved by Bioethical Committee of University of Warmia and Mazury in Olsztyn, Poland. The studies were conducted in accordance with the local legislation and institutional requirements. The participants provided their written informed consent to participate in this study. Written informed consent was obtained from the individual(s) for the publication of any potentially identifiable images or data included in this article.

## Author contributions

TS: Conceptualization, Data curation, Formal analysis, Investigation, Methodology, Project administration, Resources, Visualization, Writing – original draft. BZ: Data curation, Investigation, Resources, Writing – original draft. KJ-W: Investigation, Methodology, Project administration, Writing – original draft. KJ: Investigation, Methodology, Writing – original draft. MM: Conceptualization, Supervision, Validation, Writing – original draft. KS: Conceptualization, Funding acquisition, Methodology, Supervision, Validation, Visualization, Writing – original draft.

## References

[1] Berger J Forss-Petter S Eichler FS. Pathophysiology of X-linked adrenoleukodystrophy. Biochimie. (2014) 98:135–42. doi: 10.1016/j.biochi.2013.11.023, PMID: 24316281 PMC3988840

[2] Engelen M Kemp S Poll-The BT. X-linked adrenoleukodystrophy: pathogenesis and treatment. Curr Neurol Neurosci Rep. (2014) 14:486. doi: 10.1007/s11910-014-0486-0 25115486

[3] Moser HW Mahmood A Raymond GV. X-linked adrenoleukodystrophy. Nat Clin Pract Neurol. (2007) 3:140–51. doi: 10.1038/ncpneuro0421 17342190

[4] Cappa M Bizzarri C Vollono C Petroni A Banni S. Adrenoleukodystrophy. Endocr Dev. (2011) 20:149–60. doi: 10.1159/000321236 21164268

[5] Castellano A Papinutto N Cadioli M Brugnara G Iadanza A Scigliuolo G. Quantitative MRI of the spinal cord and brain in adrenomyeloneuropathy: in vivo assessment of structural changes. Brain. (2016) 139:1735–46. doi: 10.1093/brain/aww068 27068048

[6] Buermans NJML van den Bosch SJG Huffnagel IC Steenweg ME Engelen M Oostrom KJ. Overall intact cognitive function in male X-linked adrenoleukodystrophy adults with normal MRI. Orphanet J Rare Dis. (2019) 14:217. doi: 10.1186/s13023-019-1184-4, PMID: 31521182 PMC6744701

[7] van Geel BM Koelman JH Barth PG Ongerboer de Visser BW. Peripheral nerve abnormalities in adrenomyeloneuropathy: a clinical and electrodiagnostic study. Neurology. (1996) 46:112–8. doi: 10.1212/wnl.46.1.112 8559356

[8] Stradomska TJ Drabko K Moszczyńska E Tylki-Szymańska A. Monitoring of very long-chain fatty acids levels in X-linked adrenoleukodystrophy, treated with haematopoietic stem cell transplantation and Lorenzo's oil. Folia Neuropathol. (2014) 52:159–63. doi: 10.5114/fn.2014.43787, PMID: 25118901

[9] Shapiro E Krivit W Lockman L Jambaqué I Peters C Cowan M. Long-term effect of bone-marrow transplantation for childhood-onset cerebral X-linked adrenoleukodystrophy. Lancet. (2000) 356:713–8. doi: 10.1016/S0140-6736(00)02629-5, PMID: 11085690

[10] Kahraman S Beyazyurek C Yesilipek MA Ozturk G Ertem M Anak S. Successful haematopoietic stem cell transplantation in 44 children from healthy siblings conceived after preimplantation HLA matching. Reprod Biomed Online. (2014) 29:340–51. doi: 10.1016/j.rbmo.2014.05.010, PMID: 25066893

[11] Page KM Stenger EO Connelly JA Shyr D West T Wood S. Hematopoietic stem cell transplantation to treat Leukodystrophies: clinical practice guidelines from the Hunter's Hope Leukodystrophy care network. Biol Blood Marrow Transplant. (2019) 25:e363–74. doi: 10.1016/j.bbmt.2019.09.003, PMID: 31499213

[12] Eichler F Duncan C Musolino PL Orchard PJ de Oliveira S Thrasher AJ. Hematopoietic stem-cell gene therapy for cerebral Adrenoleukodystrophy. N Engl J Med. (2017) 377:1630–8. doi: 10.1056/NEJMoa1700554, PMID: 28976817 PMC5708849

[13] Casasnovas C Ruiz M Schlüter A Naudí A Fourcade S Veciana M. Biomarker identification, safety, and efficacy of high-dose antioxidants for Adrenomyeloneuropathy: a phase II pilot study. Neurotherapeutics. (2019) 16:1167–82. doi: 10.1007/s13311-019-00735-2 31077039 PMC6985062

[14] Shih CC Wu YR Lee-Chen GJ Chao CY. Effect of statin treatment on adrenomyeloneuropathy with cerebral inflammation: a revisit. Clin Neurol Neurosurg. (2013) 115:624–7. doi: 10.1016/j.clineuro.2012.06.024, PMID: 22795299

[15] Köhler W Engelen M Eichler F Lachmann R Fatemi A Sampson J. Safety and efficacy of leriglitazone for preventing disease progression in men with adrenomyeloneuropathy (ADVANCE): a randomised, double-blind, multi-Centre, placebo-controlled phase 2–3 trial. Lancet Neurol. (2023) 22:127–36. doi: 10.1016/S1474-4422(22)00495-1, PMID: 36681445 PMC11847323

[16] Barreca MM Cancemi P Geraci F. Mesenchymal and induced pluripotent stem cells-derived extracellular vesicles: The new frontier for regenerative medicine? Cell. (2020) 9:1163. doi: 10.3390/cells9051163, PMID: 32397132 PMC7290733

[17] Brown C McKee C Bakshi S Walker K Hakman E Halassy S. Mesenchymal stem cells: cell therapy and regeneration potential. J Tissue Eng Regen Med. (2019) 13:1738–55. doi: 10.1002/term.2914 31216380

[18] Lo Furno D Mannino G Giuffrida R. Functional role of mesenchymal stem cells in the treatment of chronic neurodegenerative diseases. J Cell Physiol. (2018) 233:3982–99. doi: 10.1002/jcp.26192, PMID: 28926091

[19] Mazzini L Vescovi A Cantello R Gelati M Vercelli A. Stem cells therapy for ALS. Expert Opin Biol Ther. (2016) 16:187–99. doi: 10.1517/14712598.2016.1116516 26558293

[20] Karussis D Kassis I Kurkalli BG Slavin S. Immunomodulation and neuroprotection with mesenchymal bone marrow stem cells (MSCs): a proposed treatment for multiple sclerosis and other neuroimmunological/neurodegenerative diseases. J Neurol Sci. (2008) 265:131–5. doi: 10.1016/j.jns.2007.05.005, PMID: 17610906

[21] Barczewska M Maksymowicz S Zdolińska-Malinowska I Siwek T Grudniak M. Umbilical cord mesenchymal stem cells in amyotrophic lateral sclerosis: an original study. Stem Cell Rev Rep. (2020) 16:922–32. doi: 10.1007/s12015-020-10016-7, PMID: 32725316 PMC7456414

[22] Struys T Moreels M Martens W Donders R Wolfs E Lambrichts I. Ultrastructural and immunocytochemical analysis of multilineage differentiated human dental pulp-and umbilical cord-derived mesenchymal stem cells. Cells Tissues Organs. (2011) 193:366–78. doi: 10.1159/000321400, PMID: 21124001

[23] Dominici M Le Blanc K Mueller I Slaper-Cortenbach I Marini F Krause D. Minimal criteria for defining multipotent mesenchymal stromal cells. The International Society for Cellular Therapy position statement. Cytotherapy. (2006) 8:315–7. doi: 10.1080/14653240600855905, PMID: 16923606

[24] Larson ST Wilbur J. Muscle weakness in adults: evaluation and differential diagnosis. Am Fam Physician. (2020) 101:95–108. PMID: 31939642

[25] Stradomska TJ Tylki-Szymańska A. Serum very-long-chain fatty acids levels determined by gas chromatography in the diagnosis of peroxisomal disorders in Poland. Folia Neuropathol. (2009) 47:306–13. PMID: 20054782

[26] Miller WP Rothman SM Nascene D Kivisto T DeFor TE Ziegler RS. Outcomes after allogeneic hematopoietic cell transplantation for childhood cerebral adrenoleukodystrophy: the largest single-institution cohort report. Blood. (2011) 118:1971–8. doi: 10.1182/blood-2011-01-329235, PMID: 21586746

[27] Yalcin K Çelen SS Daloglu H Demir MK Öztürkmen S Pasayev D. Allogeneic hematopoietic stem cell transplantation in patients with childhood cerebral adrenoleukodystrophy: a single-center experience "better prognosis in earlier stage". Pediatr Transplant. (2021) 25:e14015. doi: 10.1111/petr.14015, PMID: 33780114

[28] Ikeuchi T Fitrah YA Shu B. Loss of homeostatic microglia in rare neurological disorders: implications for cell transplantation. Nihon Yakurigaku Zasshi. (2021) 156:225–9. doi: 10.1254/fpj.21017, PMID: 34193701

[29] Cartier N Hacein-Bey-Abina S Bartholomae CC Bougnères P Schmidt M von Kalle C. Lentiviral hematopoietic cell gene therapy for X-linked adrenoleukodystrophy. Methods Enzymol. (2012) 507:187–98. doi: 10.1016/B978-0-12-386509-0.00010-7, PMID: 22365775

[30] Wu T Liu Y Wang B Li G. The roles of mesenchymal stem cells in tissue repair and disease modification. Curr Stem Cell Res Ther. (2014) 9:424–31. doi: 10.2174/1574888x09666140616125446 24998241

[31] Matysiak M Orlowski W Fortak-Michalska M Jurewicz A Selmaj K. Immunoregulatory function of bone marrow mesenchymal stem cells in EAE depends on their differentiation state and secretion of PGE2. J Neuroimmunol. (2011) 233:106–11. doi: 10.1016/j.jneuroim.2010.12.004, PMID: 21354631

[32] Drago D Basso V Gaude E Volpe G Peruzzotti-Jametti L Bachi A. Metabolic determinants of the immune modulatory function of neural stem cells. J Neuroinflammation. (2016) 13:232. doi: 10.1186/s12974-016-0667-7, PMID: 27590826 PMC5009670

[33] Guo Y Chan KH Lai WH Siu CW Kwan SC Tse HF. Human mesenchymal stem cells upregulate CD1dCD5(+) regulatory B cells in experimental autoimmune encephalomyelitis. Neuroimmunomodulation. (2013) 20:294–303. doi: 10.1159/000351450, PMID: 23899693

[34] Uccelli A Laroni A Brundin L Clanet M Fernandez O Nabavi SM. MEsenchymal StEm cells for multiple sclerosis (MESEMS): a randomized, double blind, cross-over phase I/II clinical trial with autologous mesenchymal stem cells for the therapy of multiple sclerosis. Trials. (2019) 20:263. doi: 10.1186/s13063-019-3346-z, PMID: 31072380 PMC6507027

[35] Hou ZL Liu Y Mao XH Wei CY Meng MY Liu YH. Transplantation of umbilical cord and bone marrow-derived mesenchymal stem cells in a patient with relapsing-remitting multiple sclerosis. Cell Adhes Migr. (2013) 7:404–7. doi: 10.4161/cam.26941, PMID: 24192520 PMC3903683

[36] Petrou P Kassis I Levin N Paul F Backner Y Benoliel T. Beneficial effects of autologous mesenchymal stem cell transplantation in active progressive multiple sclerosis. Brain. (2020) 143:3574–88. doi: 10.1093/brain/awaa333, PMID: 33253391

[37] Cohen JA Imrey PB Planchon SM Bermel RA Fisher E Fox RJ. Pilot trial of intravenous autologous culture-expanded mesenchymal stem cell transplantation in multiple sclerosis. Mult Scler. (2018) 24:501–11. doi: 10.1177/1352458517703802, PMID: 28381130 PMC5623598

[38] Cohen JA Lublin FD Lock C Pelletier D Chitnis T Mehra M. Evaluation of neurotrophic factor secreting mesenchymal stem cells in progressive multiple sclerosis. Mult Scler J. (2023) 29:92–106. doi: 10.1177/13524585221122156, PMID: 36113170 PMC9896300

[39] Harris VK Stark J Vyshkina T Blackshear L Joo G Stefanova V. Phase I trial of intrathecal mesenchymal stem cell-derived neural progenitors in progressive multiple sclerosis. EBioMedicine. (2018) 29:23–30. doi: 10.1016/j.ebiom.2018.02.002, PMID: 29449193 PMC5925446

[40] Spees JL Lee RH Gregory CA. Mechanisms of mesenchymal stem/stromal cell function. Stem Cell Res Ther. (2016) 7:125. doi: 10.1186/s13287-016-0363-7, PMID: 27581859 PMC5007684

[41] Koç ON Peters C Aubourg P Raghavan S Dyhouse S DeGasperi R. Bone marrow-derived mesenchymal stem cells remain host-derived despite successful hematopoietic engraftment after allogeneic transplantation in patients with lysosomal and peroxisomal storage diseases. Exp Hematol. (1999) 27:1675–81. doi: 10.1016/s0301-472x(99)00101-0, PMID: 10560915

[42] Moser HW Moser AB Naidu S Bergin A. Clinical aspects of adrenoleukodystrophy and adrenomyeloneuropathy. Dev Neurosci. (1991) 13:254–61. doi: 10.1159/000112170 1817030

